# Mesenteric Lymphatic B Cells Migrate to the Gut and Aggravate TNBS-Induced Rat Colitis via Regulating Intestinal T Cells

**DOI:** 10.3390/ijms26083519

**Published:** 2025-04-09

**Authors:** Yu Zhang, Qinghe Zhao, Zhe Wu, Ning Chen, Na Li, Jiao Liu, Menglei Zhang, Shuolei Li, Yujing Chi, Yulan Liu

**Affiliations:** 1 Department of Gastroenterology, Peking University People’s Hospital, Beijing 100044, China; zhangyu_zxy@bjmu.edu.cn (Y.Z.); 2211210258@stu.pku.edu.cn (Q.Z.); samplezhewu@bjmu.edu.cn (Z.W.); chenning79@bjmu.edu.cn (N.C.); 2 Clinical Center of Immune-Mediated Digestive Diseases, Peking University People’s Hospital, Beijing 100044, China; 3 Department of Central Laboratory and Institute of Clinical Molecular Biology, Peking University People’s Hospital, Beijing 100044, China; yingshw@163.com; 4 Center of Medical and Health Analysis, Peking University Health Science Center, Beijing 100191, China; liujiao@bjmu.edu.cn; 5 Department of Animal Laboratory, Peking University People’s Hospital, Beijing 100044, China; zhangmenglei@pkuph.edu.cn (M.Z.); lishuolei@pkuph.edu.cn (S.L.)

**Keywords:** inflammatory bowel disease, Crohn’s disease, mesenteric lymphatics, B cells, T cells

## Abstract

Inflammatory bowel disease (IBD), comprising Crohn’s disease (CD) and ulcerative colitis (UC), is affecting a growing global population. Unlike UC, which is characterized by inflammation confined to the intestinal mucosa and submucosa, CD involves transmural inflammation of the intestine. Although the lymphatic system is believed to play a role in the pathogenesis of CD, its exact contribution remains poorly understood. Mesenteric lymphatics (MLs), which drain interstitial fluid and immune cells into mesenteric lymph nodes, have been implicated in this process. In the present study, we aimed to investigate the role of ML immune cells in TNBS-induced colitis in rats. Flow cytometry analysis revealed an increased ratio of B cells and altered B cell function in the MLs of colitis rats compared to controls. The adoptive transfer of mesenteric lymphatic B (MLB) cells isolated from colitis rats to recipient rats exacerbated colitis and was associated with the enhanced migration of MLB cells to the gut. RNA sequencing analysis demonstrated a significant upregulation of genes associated with inflammation and immune responses in MLB cells from colitis rats, particularly key molecules involved in T cell activation, such as *cluster of differentiation 27* (*Cd27*) and *cluster of differentiation 40* (*Cd40*), and the chemotactic receptor *C-C motif chemokine receptor 8* (*Ccr8*), which mediates B cell migration in response to T cells. Mechanistically, MLB cells from colitis rats were recruited to the colon by intra-intestinal T cells through the Ccr8-C-C motif chemokine ligand 1 (Ccl1) axis, where they subsequently exacerbated inflammatory responses via enhanced differentiation. These observations indicate that the migration of MLB cells to the gut exacerbates TNBS-induced colitis in rats by modulating intestinal T cells.

## 1. Introduction

Inflammatory bowel disease (IBD), which includes Crohn’s disease (CD) and ulcerative colitis (UC), is affecting a growing global population. The etiology of IBD involves a complex interplay of factors, including environmental influences, genetic susceptibility, intestinal microbiota, and lifestyle, all of which contribute to an excessive immune response and chronic inflammation. Unlike the limited inflammation confined to the intestinal mucosa and submucosa in UC, CD is characterized by transmural inflammation of the intestine, which is accompanied by an increased density of lymphatic vessels in both the intestine and mesentery [1]. Although the role of immune response dysfunction in IBD is well established, the involvement of the lymphatic system in the pathogenesis of CD remains poorly understood.

Mesenteric lymph nodes (MLNs) and tertiary lymphoid organs (TLOs) are essential components of gut-associated lymphoid tissue (GALT). Several studies have demonstrated a positive correlation between the number of regional lymph nodes and the severity of jejunal CD [2]. Additionally, the size of MLNs is found to correlate with intestinal histological changes in CD [3], and enlarged MLNs have been identified as independent predictors of CD recurrence [4]. In addition to MLNs, TLOs resemble secondary lymphoid organs and function as valves in lymphatic fluid flow, serving as hubs for the collection of cellular and molecular components from the gut to the mesenteric lymphatics (MLs) [5]. In patients with CD, numerous TLOs have been observed within the collecting lymphatic vessels in the mesentery adjacent to the inflamed ileum [6]. The administration of tumor necrosis factor-α (TNF-α) inhibitors has been shown to restore the flow of intestinal substances obstructed by TLOs into the lymphatic vessels and to alleviate intestinal inflammation in ileitis mouse models [7]. These findings suggest that lymphatic vessel function plays a critical role in the pathogenesis of CD and is closely associated with MLNs and TLOs.

Lymphatic capillaries in the intestine, located within the lamina propria, submucosa, and muscularis layers, play a crucial role in draining interstitial fluid and immune cells into the MLs and MLNs and ultimately back into the bloodstream. Studies have shown that lymphatic vessel density is increased in both the inflamed and non-inflamed intestinal mucosa of patients with CD compared to healthy controls [1]. Furthermore, morphological changes in MLs, such as lymphatic congestion, expansion, remodeling, and lymphangiogenesis, have been observed in patients with CD [8]. In addition to their role in transporting fluids, salts, fats, and nutrients from the intestine, MLs also facilitate immune cell trafficking [9]. The dysfunction of these vessels disrupts immune cell migration and impairs downstream immune regulation. Notably, lymphangitis has been reported since CD was first recognized as a disease [10]. It is indicated that the composition of lymphatic fluid changes in response to intestinal inflammation; however, studies specifically investigating these alterations in CD remain limited.

Dendritic cells (DCs) in the intestinal mucosa recognize intestinal antigens and migrate through MLs to MLNs, where they initiate the activation of B cells and T cells. The activated B and T cells then migrate back to the intestine, where they fulfill their immune functions [11]. DCs present antigens to B and T cells, suggesting that adaptive immune cells may also undergo changes within the lymphatic system. Several studies have reported the increased migration of DCs from the lamina propria to MLNs via the collection of lymphatics in UC and inflammation diseases [12,13]. However, the role of other adaptive immune cells in lymphatic fluid remains poorly understood. Notably, significant B cell activity and evident TLO invasion into lymphatic vessels within the mesenteric tissues of patients with CD have been observed [6], suggesting that B cells may play a specific role in the pathogenesis of CD. However, it remains unclear whether these B cells originate from the MLs.

In this study, a TNBS-induced colitis rat model was used to investigate the role of mesenteric lymphatic B (MLB) cells in CD through adoptive transfer experiments, with a particular focus on the role of MLB cells on intestinal T cells.

## 2. Results

### 2.1. The Ratio of B Cells Increased in ML and MLN in TNBS-Induced Colitis Rats

**Figure 1:**
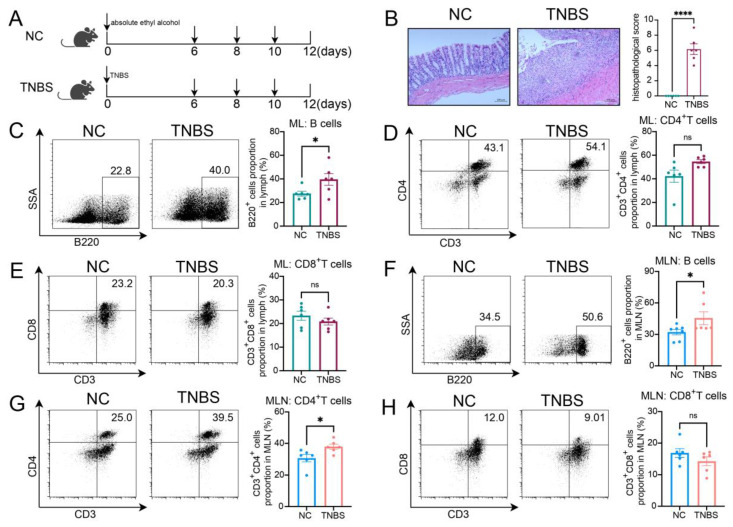
The ratio of B cells increased in MLs and MLNs in TNBS-induced colitis rats. (**A**) Model of TNBS-induced acute colitis in rats. (**B**) H&E staining images from NC and TNBS rats are presented on the left, with quantitative analysis provided on the right. Representative flow cytometry images and statistical analysis of B cells (**C**), CD4^+^ T cells (**D**), and CD3^+^CD8^+^ T cells (**E**) in the MLs of two groups. Representative flow cytometry images and statistical analysis of B cells (**F**), CD4^+^ T cells (**G**), and CD3^+^CD8^+^ T cells (**H**) in the MLNs of two groups. NC—normal control rats; TNBS—TNBS-induced colitis rats; MLs—mesenteric lymphatics; MLNs—mesenteric lymph nodes. N = 6. * *p* < 0.05; **** *p* < 0.0001 with NC rats; n.s.—no significant difference.

Rats were administrated TNBS to induce CD, as shown in Figure 1A. Colonic histopathological analysis confirmed that colitis was successfully induced (Figure 1B). Compared with the normal control (NC) group, TNBS-induced colitis rats exhibited a higher ratio of B cells within the mesenteric lymphatics (Figure 1C), while there was no significant difference in the proportions of CD3^+^CD4^+^ T cells and CD3^+^CD8^+^ T cells between the two groups (Figure 1D,E). In the MLNs of the rats, B cells were also increased in the TNBS group (Figure 1F), along with CD3^+^CD4^+^ T cells (Figure 1G), while the ratio of CD3^+^CD8^+^ T cells remained unchanged (Figure 1H). These findings revealed an increase in B cells within both the MLs and MLNs of TNBS-induced colitis rats, suggesting that B cells may play a significant role in the pathogenesis of CD.

### 2.2. MLB Cells Exhibit a Tendency to Migrate to the Intestine in TNBS-Induced Colitis Rats

**Figure 2:**
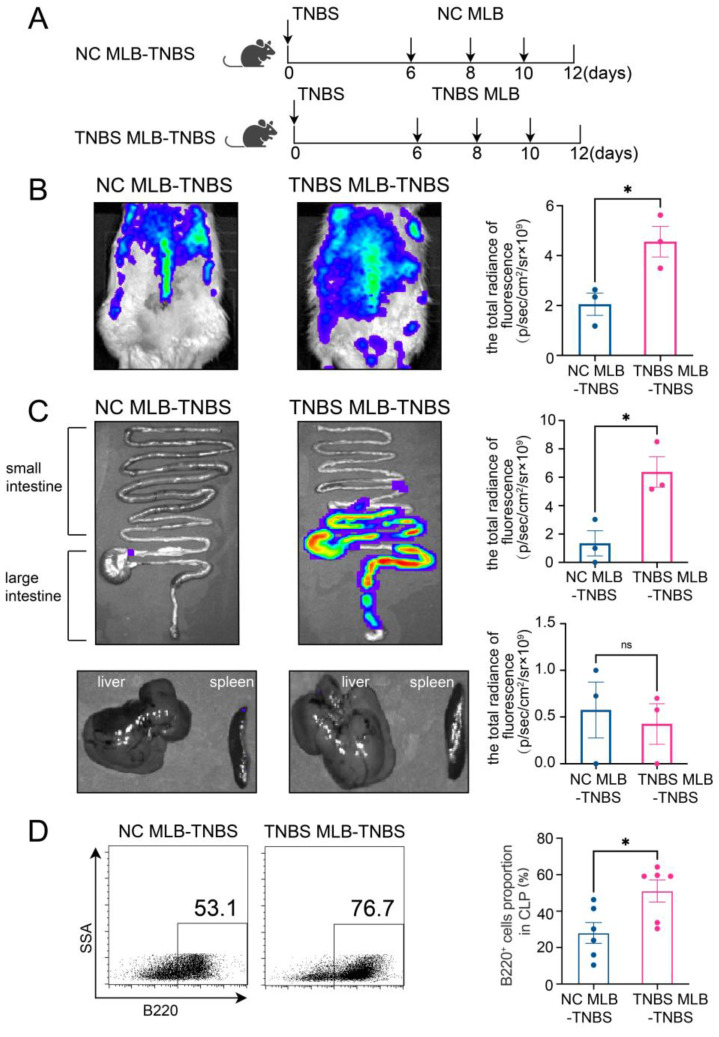
(**A**) Model of adoptive transfer of MLB cells to TNBS-induced colitis rats. A total of 5 × 10^6^ NC MLB or TNBS MLB cells labeled with 1,1-dioctadecyl-3,3,3,3-tetramethylindotricarbocyaineiodide (DiR) were administered to colitis rats through adoptive transfer on three separate occasions. Representative fluorescence images of the rat (**B**), intestine (**C**), and liver and spleen (**D**) of the NC MLB-TNBS group and the TNBS MLB-TNBS group are shown on the left, with statistical comparisons displayed on the right at 24 h post-transfer. The pseudocolor representation depicts the fluorescence intensity following adoptive transfer of MLB cells, with the color gradient ranging from blue (low intensity) to red (high intensity). (**E**) Flow cytometry analysis results for the labeled B cells in the intestinal lamina propria of rats that received MLB cells from colitis or NC rats. NC MLB—MLB cells derived from the normal control rat; TNBS MLB—MLB cells derived from TNBS-induced colitis rat; NC MLB-TNBS—TNBS-induced colitis rats receiving NC MLB cells; TNBS MLB-TNBS—TNBS-induced colitis rats receiving TNBS MLB cells. N = 3–6. * *p* < 0.05; n.s.—no significant difference.

**Figure 3:**
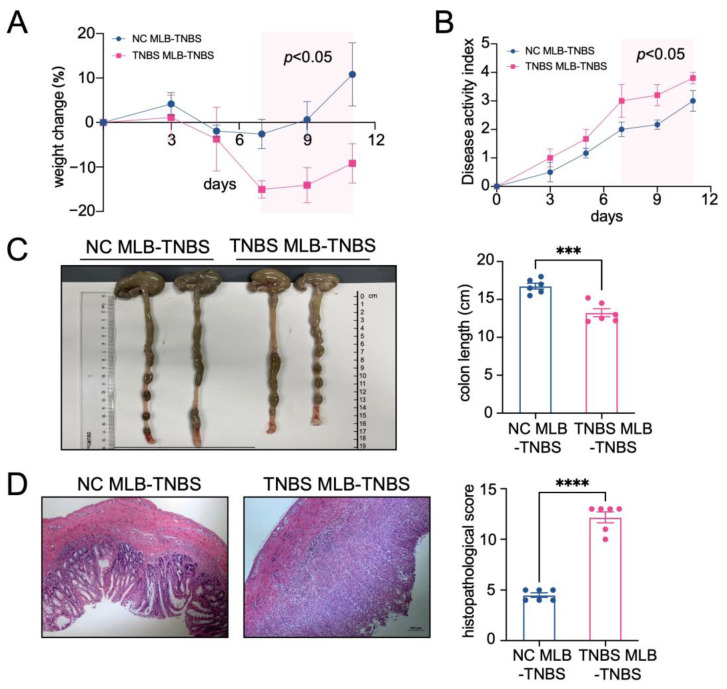
The adoptive transfer of TNBS-induced MLB cells aggravated colitis. A weight change (**A**) and disease activity index (**B**) comparison between the NC MLB-TNBS group and the TNBS MLB-TNBS group. The shaded area indicates the time period during which a statistically significant difference (*p* < 0.05) was observed between the two groups (**C**,**D**) Representative colon-length and intestinal pathological images are shown on the left, with statistical analysis shown on the right. NC MLB-TNBS—TNBS-induced colitis rats receiving NC MLB cells; TNBS MLB-TNBS—TNBS-induced colitis rats receiving TNBS MLB cells. N = 6. *** *p* < 0.001; **** *p* < 0.0001.

Furthermore, to investigate the migratory potential of MLB cells to the intestinal tract, we employed CD45RA microbeads to isolate MLB cells from both control and TNBS-induced colitis rats. These isolated cells were subsequently administered to recipient rats with colitis through tail vein injection (Figure 2A). As shown in Figure 2B, recipient rats that received MLB cells from colitis rats displayed increased fluorescence intensity in the abdominal region when compared to **rats** receiving MLB cells derived from NC rats. Additionally, organs, including the intestine, liver, and spleen, were extracted and analyzed using intravital imaging. The results demonstrated that rats receiving MLB cells from TNBS-induced colitis rats exhibited increased fluorescence intensity in the intestine but not in the liver or spleen (Figure 2C,D). Consistent with these findings, flow cytometry analysis revealed an increased presence of labeled B cells in the intestinal lamina propria of rats that received MLB cells from rats with TNBS-induced colitis (Figure 2E). Following this, the progression of colitis was evaluated after adoptive transfer. Rats receiving MLB cells from the colitis group showed more severe colitis compared to those receiving MLB cells from the control group, including greater weight loss (Figure 3A), higher disease activity indexes (Figure 3B), shorter colon lengths (Figure 3C), and higher histopathological inflammatory scores (Figure 3D). These findings demonstrate that MLB cells exhibit a strong tropism toward the intestinal tract and potentially contribute to the exacerbation of intestinal inflammation in TNBS-induced colitis models.

### 2.3. MLB Cells Migrate to Colon by Intestinal T Cells Through Ccr8-Ccl1 Axis

**Figure 4:**
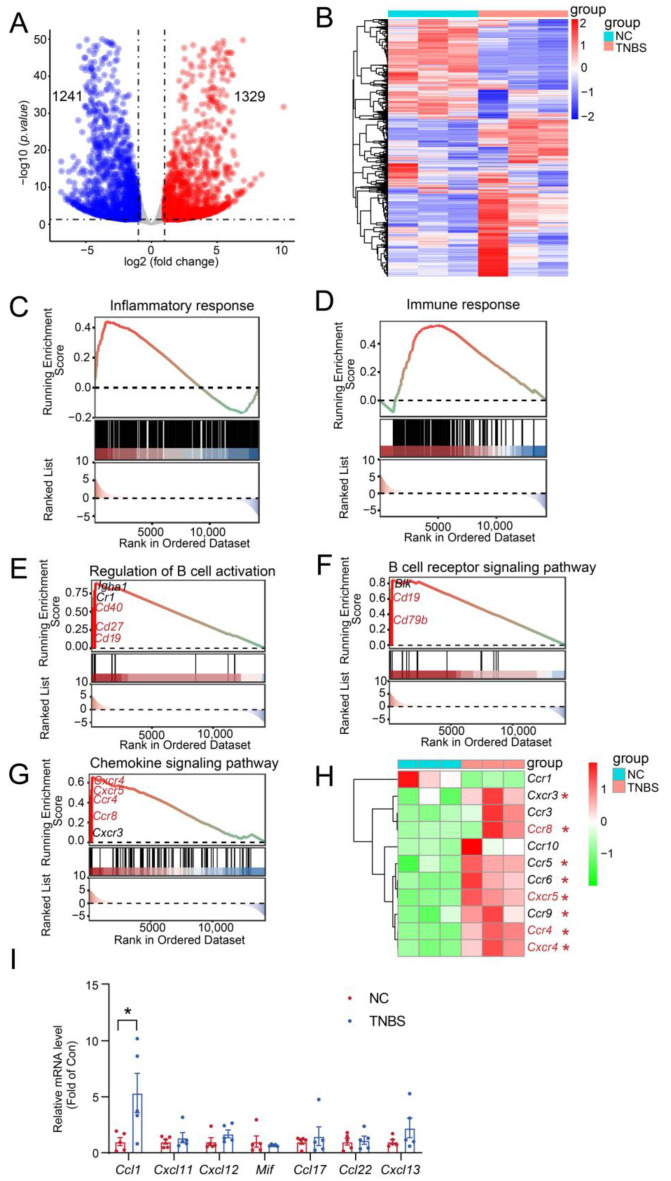
Inflammatory and immune responses were activated in MLB cells with colitis. The volcano plot analysis (**A**) and heatmap (**B**) visualize the transcriptional differences between MLB cells from the NC and TNBS groups. In the volcano plot, red dots represent significantly upregulated transcripts, and blue dots represent significantly downregulated transcripts. Dashed lines indicate the thresholds for differential expression: log_2_(fold change) > 1 or < –1 (vertical) and adjusted *p*-value < 0.05 (horizontal). Pathway enrichment highlights core genes upregulated in B cell-driven inflammatory responses (**C**), B cell-mediated immune response (**D**), the regulation of B cell activation (**E**), the B cell receptor signaling pathway (**F**), and the B cell chemokine signaling pathway (**G**) between the MLB cells of NC and TNBS. The red-to-green color gradient indicates the ranking of genes from high to low expression. The horizontal dotted line in the upper panel represents the zero enrichment score, and the dotted line in the lower panel indicates the baseline of the ranking metric. (**H**) The heatmap displays the different chemokine-related genes between the MLB cells of NC and TNBS. (**I**) Comparison of the mRNA expression of *Ccl1* (ligand of Ccr8), *Mif*, *Cxcl12*, and *Cxcl11* (ligands of Cxcr4), *Ccl17* and *Ccl22* (ligands of Ccr4), and *Cxcl13* (ligand of Cxcr5) in colonic tissues between NC and TNBS rats. MLB—mesenteric lymphatic B cells; NC—normal control rats; TNBS—TNBS-induced colitis rats. Red asterisk represents upregulated transcripts. N = 3–5. * *p* < 0.05.

To better understand the involvement of MLB cells in TNBS-mediated colitis, RNA-seq analysis was carried out on MLB cells derived from both NC and TNBS-induced colitis rats. We identified 2570 significant transcriptional changes, with 1329 transcripts upregulated and 1241 transcripts downregulated in MLB cells derived from TNBS-induced colitis rats, compared to NC rats (Figure 4A,B). Gene Set Enrichment Analysis (GSEA) further demonstrated that inflammatory and immune responses were significantly activated in MLB cells from TNBS-induced colitis rats, compared to those from NC rats (Figure 4C,D). Additionally, significant enrichment was observed in genes associated with the regulation of B cell activation (*cluster of differentiation 19* [*Cd19*], *Cd27*, *Cd40*) (Figure 4E), the B cell receptor signaling pathway (*Cd19*, *Cd79b*) (Figure 4F), and the chemokine signaling pathway (*C-X-C motif chemokine receptor 4* [*Cxcr4*], *Cxcr5*, *C-C motif chemokine receptor* [*Ccr4*], *Ccr8*) (Figure 4G). Consistent with the GSEA results, the mRNA expression of B cell-related chemotactic receptors, including *Cxcr4*, *Cxcr5*, *Ccr4*, and *Ccr8*, was significantly increased in MLB cells derived from TNBS-induced rats in comparison with NC rats (Figure 4H). To assess whether the upregulation of chemokine receptors in MLB cells corresponds to chemokine signals in the inflamed colon, we examined the mRNA expression of their respective ligands in colonic tissues from TNBS-induced colitis rats and normal controls. Among these, the mRNA expression of *C-C motif chemokine ligand 1* (*Ccl1*), the ligand of Ccr8, was significantly upregulated in the colon of TNBS rats (Figure 4I). In contrast, the mRNA expression of ligands for Cxcr4 (*macrophage migration inhibitory factor* [*Mif*], *C-X-C motif chemokine ligand 12* [*Cxcl12*], *Cxcl11*), Cxcr5 (*Cxcl13*), and Ccr4 (*Ccl17*, *Ccl22*) showed no significant differences or only mild, non-significant trends in the colon (Figure 4I). These results suggested that the Ccr8–Ccl1 axis may play a predominant role in mediating MLB cell migration to the inflamed intestine.

**Figure 5:**
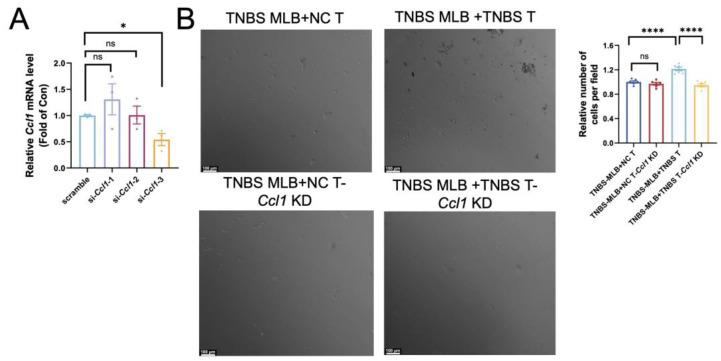
Effect of *Ccl1* silencing in colonic CD4^+^ T cells on migratory behavior of MLB cells. (**A**) Relative *Ccl1* mRNA expression after si-*Ccl1*-1, -2, or -3 treatment was assessed, with *p*-values of 0.3566, 0.9593, and 0.0174, respectively. (**B**) Representative images of cells in the lower chamber are shown for the TNBS MLB+NC T group, TNBS MLB+NC T-*Ccl1* KD group, TNBS MLB+TNBS T group, and TNBS MLB+TNBS T-*Ccl1* KD group in the left panel, and statistical analysis for the four groups is displayed in the right panel. TNBS MLB+NC T—co-culture of mesenteric lymphatic B cells from TNBS rats with colonic CD4^+^ T cells from NC rats; TNBS MLB+NC T-*Ccl1* KD—co-culture of mesenteric lymphatic B cells from TNBS rats with *Ccl1*-knockdown colonic CD4^+^ T cells from NC rats; TNBS MLB+TNBS T—co-culture of mesenteric lymphatic B cells from TNBS rats with colonic CD4^+^ T cells from TNBS-induced colitis rats; TNBS MLB+TNBS T-*Ccl1* KD—co-culture of mesenteric lymphatic B cells from TNBS rats with *Ccl1*-knockdown colonic CD4^+^ T cells from TNBS-induced colitis rats. N = 3–6. * *p* < 0.05; **** *p* < 0.0001; n.s.—no significant difference.

Extensive research has established the pivotal role of intra-intestinal T cells in mediating intestinal inflammation during colitis. Since Ccl1 serves as a ligand on T cells for Ccr8, we performed RNA interference to suppress *Ccl1* expression in colonic CD4^+^ T cells. Among the siRNAs tested, si-*Ccl1-3* achieved the most effective silencing, reducing *Ccl1* mRNA levels by approximately 60–70%, and was therefore chosen for use in the subsequent assays (Figure 5A). Colonic CD4^+^ T cells isolated from normal control rats and TNBS-induced colitis rats were seeded into the lower chamber of a Transwell system and treated with either control siRNA or si-*Ccl1-3* for 24 h. Subsequently, MLB cells sorted from TNBS rats were placed in the upper chamber and co-cultured for an additional 24 h. Due to the similar morphology and size of CD4^+^ T and B cells, migrated MLB cells could not be directly distinguished from resident CD4^+^ T cells. Therefore, the total number of cells in the lower chamber was quantified as an indirect indicator of MLB cell migration. The total number of migrated cells was significantly higher when both MLB cells and T cells were obtained from TNBS-induced colitis rats compared to experiments using MLB cells from colitis rats with T cells from NC rats (Figure 5B). This suggests that TNBS-derived T cells exhibit a stronger chemotactic effect on MLB cells. Additionally, *Ccl1* knockdown in T cells from TNBS colitis rats significantly reduced the number of migrated MLB cells, whereas *Ccl1* knockdown in T cells from NC rats showed no significant effect (Figure 5B). These findings suggest that T cells from TNBS-induced colitis rats promote MLB cell recruitment through the Ccr8–Ccl1 axis during intestinal inflammation.

### 2.4. MLB Cells Aggravate Colitis by Inducing Intestinal T Cell Differentiation

**Figure 6:**
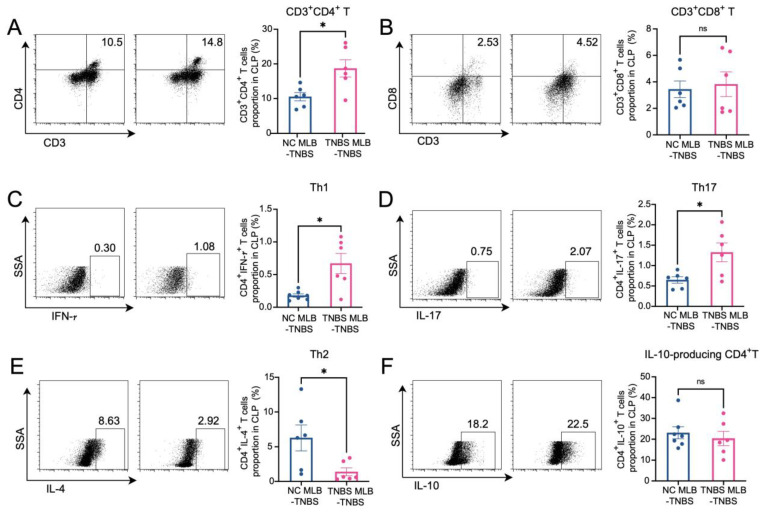
Alterations in T cells in colonic lamina propria. The figures show the ratio of CD3^+^CD4^+^ T cells (**A**), CD3^+^CD8^+^T cells (**B**), CD4^+^IFN-γ^+^ T cells (Th1) (**C**), CD4^+^IL-17^+^ T cells (Th17) (**D**), CD4^+^IL-4^+^ T cells (Th2) (**E**), and IL-10-producing CD4^+^ cells (**F**) in the colonic lamina propria of the NC MLB-TNBS and TNBS MLB-TNBS groups. NC MLB-TNBS—TNBS-induced colitis rats administered MLB cells from NC rats; TNBS MLB-TNBS—TNBS-induced colitis rats administered MLB cells from TNBS rats. N = 6. * *p* < 0.05; n.s.—no significant difference.

**Figure 7:**
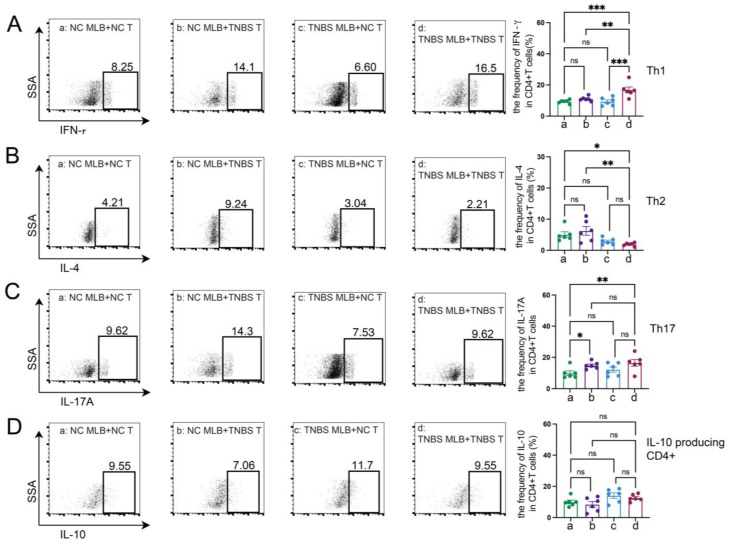
MLB cells regulated the differentiation of T cells in vitro. The figures show the ratio of CD4^+^IFN-γ^+^ T cells (Th1) (**A**), CD4^+^IL-4^+^ T cells (Th2) (**B**), CD4^+^IL-17^+^ T cells (Th17) (**C**), and IL-10-producing CD4^+^ cells (**D**) in the co-culture experiments of MLB cells and intestinal CD4^+^ T cells. NC MLB—mesenteric lymphatic B cells derived from normal control rats; NC T—CD4^+^ T cells derived from colon of normal control rats; TNBS T—CD4^+^ T cells derived from colon of TNBS-induced colitis rats; TNBS MLB—mesenteric lymphatic B cells isolated from TNBS-induced colitis rats. N = 6. Statistical *p*-values are marked in each panel; * *p* < 0.05, ** *p* < 0.01, *** *p* < 0.001, n.s.—no significant difference.

The RNA sequencing of MLB cells revealed an upregulation of mRNA levels of *Cd27* and *Cd40*, key molecules involved in T cell activation. Based on these findings, we further investigated the effects of MLB cells on intestinal T cells. Compared to colitis rats that received MLB cells from the control group, those that received MLB cells from the colitis group exhibited higher levels of CD3^+^CD4^+^ T cells, while they had little effect on CD3^+^CD8^+^ T cells (Figure 6A,B). Furthermore, MLB cells derived from TNBS-induced colitis rats significantly increased the proportions of CD4^+^ interferon-γ^+^ (IFN-γ^+^) T (Th1) cells (Figure 6C) and CD4^+^interleukin-17^+^ (IL-17^+^) T (Th17) cells (Figure 6D) in the colonic lamina propria, while simultaneously reducing the proportion of CD4^+^IL-4^+^ T (Th2) cells, compared to the control group (Figure 6E). However, no significant differences were observed in the levels of IL-10-producing CD4^+^ T cells between the two groups (Figure 6F). These findings indicate that MLB cells from colitis rats promote a T cell-related inflammatory response. To further confirm the role of MLB cells in T cell regulation, co-culture experiments were conducted. MLB cells isolated from NC rats had no effect on the differentiation of Th1/Th2/Th17/IL-10-producing CD4^+^ cells, regardless of whether these CD4^+^ T cells were derived from NC or colitis groups (Figure 7A–D). Similarly, MLB cells isolated from either control or TNBS-induced colitis rats showed no significant effect on the differentiation of intestinal CD4^+^ T cells derived from control rats, as demonstrated by the comparable proportions of Th1, Th2, Th17, and IL-10-producing CD4^+^ cell subsets. However, MLB cells isolated from TNBS-induced colitis rats significantly promoted the differentiation of intestinal CD4^+^ T cells from colitis rats into Th1 cells (Figure 7A) while inhibiting their differentiation into Th2 cells (Figure 7B), consistent with the findings observed in the adoptive transfer experiments of TNBS-induced MLB cells into colitis rats (Figure 6C,E). Notably, MLB cells isolated from TNBS-induced colitis rats specifically enhanced the Th1 proportion in colitis-derived CD4^+^T cells but had no effect on control CD4^+^T cells (Figure 7A). Furthermore, no significant alterations were observed in the differentiation of CD4^+^ T cells into Th17 or IL-10-producing CD4^+^ cell subsets (Figure 7C,D). Previous studies have demonstrated that CD is a Th1-predominant disorder, and modulating the differentiation of Th1 cells toward Th2 cells can ameliorate colitis induced by TNBS in rats [14,15]. Collectively, these findings demonstrate that the colitis-exacerbating potential of MLB cells is primarily mediated by their ability to promote Th1 differentiation while suppressing Th2 differentiation in the intestine.

## 3. Discussion

**Figure 8:**
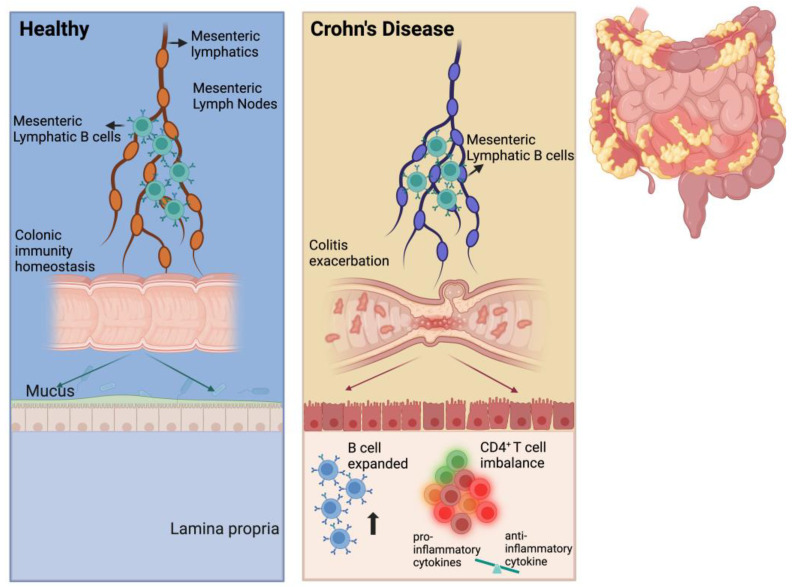
Scheme of MLB cells aggravating colitis. We propose that the number of MLB cells increases in TNBS-induced colitis rats and that these cells migrate to the intestine. In the intestine, the number of B cells also increases, promoting the differentiation of intestinal T cells into pro-inflammatory subtypes, eventually resulting in colitis exacerbation.

In the present study, we found that MLB cells were increased in TNBS-induced colitis in rats. The adoptive transfer of MLB cells isolated from colitis rats to recipient rats exacerbated colitis, which was associated with the increased migration of MLB cells to the intestine. RNA sequencing analysis revealed a significant upregulation of genes associated with inflammation and immune responses in MLB cells from colitis rats, particularly key molecules involved in T cell activation (*Cd27* and *Cd40*) and the chemotactic receptor (*Ccr8*), which indicates that B cells might migrate to the intestine in response to T cells. Further investigation demonstrated that MLB cells from colitis rats were recruited to the colon by intra-intestinal T cells through the Ccr8-Ccl1 axis, where they subsequently exacerbated inflammatory responses via enhanced differentiation (Figure 8).

It is widely accepted that mesenteric adipose tissue expansion plays a role in CD. Zhu et al. found that mesenteric lymphatics were discontinuous, with ruptured and incomplete lymphatic endothelial cells found in patients with CD, as well as the decreased expression of tight junction proteins. These alterations led to lymph leakage, which stimulated the inflammation and proliferation of mesenteric adipose tissue [16]. Given that lymphangiogenesis is regulated by lymphatic vascular endothelial growth factors (VEGFs) binding to VEGFR-3, the stimulation of VEGFR-3 aggravated colitis, while the administration of VEGF-C alleviated colitis [17,18,19]. Notably, mesenteric lymphatics-targeting drugs promote lymphatic drainage and improve intestinal inflammation in colitis rats [20]. In addition, given the critical role of mesenteric lymphatics in bridging intestinal antigens and MLNs, as well as the immune characteristics of colitis, we investigated the immune cells within mesenteric lymphatics, which contain various types of cells and nutrients that differ between healthy and colitis groups [21]. In the present study, an increased presence of B cells in MLs and MLNs was observed in the TNBS-induced colitis rats. Elevated B cell levels have been observed in mesenteric adipose tissue, tertiary TLOs, and MLNs of patients with CD [22,23], as well as in ileal Peyer’s patches and the appendiceal orifice of GALT samples from patients with IBD [24]. However, studies focusing on the effects of MLB cells in CD remain limited. It has been observed that B cells and prominent TLOs infiltrate lymphatic vessels in the mesenteric tissue of patients with CD, potentially impairing lymphatic drainage to MLNs [6]. Previous clinical trials targeting circulating B cells indiscriminately have proven unsuccessful or even detrimental in IBD [25,26], which may be attributed to the distinct roles of tissue-resident B cells compared to circulating B cells, as rituximab did not affect gut-resident B cells [27]. In the present study, we observed a strong propensity of MLB cells to migrate to the intestine and exacerbate colitis in rats through the adoptive transfer of MLB cells from TNBS-induced colitis rats, suggesting that resident MLB cells may contribute critically to the pathogenesis of colitis, particularly in CD.

Resident T cells have long been considered pivotal in the intestinal inflammation of CD [28]. In the present study, we found increased mRNA expression of genes related to inflammatory and immune responses, including *Cd27* and *Cd40*, as well as the chemotactic receptor Ccr8, in MLB cells from colitis rats. Additionally, changes were observed in colonic T cell proportions along with the worsening of colitis driven by MLB adoptive transfer. MLB cells promoted the differentiation of T cells, particularly enhancing Th1 cell differentiation while inhibiting Th2 cell differentiation in the intestine, leading to the development of pro-inflammatory subtypes both in vivo and in vitro. Consistent with our findings, previous studies have demonstrated that CD is a Th1-predominant disorder, while UC is considered a Th2-dominant disease characterized by Th2 cells [14]. Moreover, modulating the differentiation of Th1 cells toward Th2 cells can ameliorate colitis induced by TNBS in rats [15]. Additionally, Gadjalova et al. reported that B cells could enhance pro-inflammatory cytokine production by intestinal CD4^+^ T cells through the co-stimulatory interaction of CD40L and CD86 [29]. The depletion of B cells reduced intestinal inflammation by inhibiting pro-inflammatory cytokine production in CD4^+^ T cells in a chronic colitis mouse model [29]. IL-10-secreting B cells, a regulatory B cell subtype, have been shown to ameliorate T cell-mediated colitis [30].

In addition to affecting intestinal T cells, several studies have indicated that migrating B cells can also influence intestinal epithelial cells, thereby directly participating in intestinal barrier function. Marijana Basic et al. demonstrated that B cells can directly impact epithelial and intestinal barrier function via a CD14 infectious colitis animal model [31]. Another study highlighted the direct role of intestinal B cells in epithelial damage in IBD [32]. In our previous study, we observed the migration of MLB cells to the intestine in a dextran sodium sulfate (DSS)-induced UC animal model. Given the pivotal roles of chemokines and their ligands in inducing the migration of immune cells to sites of intestinal inflammation, we demonstrated that the migration of MLB cells was induced by the CXCL13–CXCR5 axis by the intestinal epithelium [33]. Several studies have shown that CXCL13 and CXCR5 are essential for B cell homing to secondary lymphoid organs [34,35]. Impaired Peyer’s patches and decreased lymph nodes were observed in CXCL13^−/−^ and CXCR5^−/−^ mice [34]. Although the present study did not detect alterations in CXCL13 and CXCR5 expression in MLB cells from TNBS-induced colitis rats, we observed upregulated mRNA expression of *Ccr8*, a receptor for the T cell-derived chemokine Ccl1, in these rats. Furthermore, the increased mRNA expression of *Ccl1* was also observed in the colon of TNBS-induced colitis rats. These findings imply that the chemotaxis of MLB cells and intestinal T cells may contribute to the pathogenesis of CD. Additionally, *Ccl1* knockdown in T cells from TNBS colitis rats significantly reduced the number of migrated MLB cells, whereas *Ccl1* knockdown in T cells from NC rats showed no significant effect. These findings suggest that T cells from TNBS-induced colitis rats promote MLB cell recruitment through the Ccr8–Ccl1 axis during intestinal inflammation.

Although the TNBS-induced colitis model is an acute chemically induced inflammatory model, it is widely used in IBD research due to its ability to recapitulate several key pathological features of Crohn’s disease, including mucosal inflammation, epithelial barrier disruption, and Th1-type immune responses. Its reproducibility, ease of use, and capacity to induce localized transmural inflammation make it a widely accepted model for studying intestinal immune regulation and immune cell trafficking [36]. In the present study, this model served as a reliable experimental framework, allowing us to investigate the functional roles and migratory behavior of MLB cells during intestinal inflammation. However, it does not fully reproduce the chronic, relapsing nature of human CD or the complex immunological, microbial, and genetic interactions underlying the disease [37,38]. Future studies using chronic or spontaneous models of colitis, genetic mouse models [39], or patient-derived samples are needed to further validate the pathogenic role of MLB cells and the chemokine–receptor interactions observed in this study.

Our study has several limitations that should be acknowledged. First, we did not differentiate between B cell subtypes, despite the well-documented opposing roles of B2 cells and regulatory B cells in inflammation and immune regulation. Second, B cells were transferred to recipient rats via tail vein injection, a method that may not fully recapitulate the natural migration patterns of MLB cells in vivo. Finally, the precise effects of B cells on T cells are still not completely understood, and further experimental evidence is needed to substantiate these interactions.

## 4. Materials and Methods

### 4.1. Rats and Induction of Colitis

All experimental procedures involving animals were conducted in accordance with the guidelines established by the Institutional Animal Care and Use Committee of Peking University People’s Hospital (Approval Nos. 2019PHE006; 2020PHE025; 2022PHE143). Male Sprague Dawley (SD) rats (wild type, 6–8 weeks old) were acquired from Beijing Huafukang Biotechnology Co., Ltd., Beijing, China and housed under specific pathogen-free conditions within the institutional animal facility. Animals were maintained in groups of six per cage under controlled environmental conditions (ambient temperature: 18–23 °C; relative humidity: 40–60%; 12/12 h light/dark cycle). To establish the colitis model, a 2,4,6-trinitrobenzenesulfonic acid–ethanol solution (5% TNBS dissolved in 50% ethanol, *v*/*v*) was prepared at a dosage of 100 mg/kg body weight (Sigma, St. Louis, MO, USA). The solution was administered via intrarectal instillation at a depth of 8 cm using a polyethylene catheter. Control animals received an equivalent volume of absolute ethanol through identical administration procedures. Disease progression was evaluated through the daily monitoring of body weight variations, diarrheal symptoms, and fecal occult blood detection in all experimental subjects.

### 4.2. Collecting Mesenteric Lymph

Under isoflurane-induced anesthesia (3–5% vapor concentration), animals were positioned in dorsal recumbency on a thermostatically controlled surgical platform maintained at 37 °C. Following trichotomy of the midline abdominal region, sequential antisepsis was performed using povidone–iodine solution followed by 70% ethanol (*v*/*v*). A paramedian laparotomy (2–4 cm in length) was executed using microsurgical scissors, extending through the cutaneous and muscular layers. The intestinal tract was exteriorized and stabilized against the left abdominal wall with saline-moistened sterile gauze. Subsequent microsurgical dissection identified the mesenteric lymphatic duct (0.5–1 mm diameter) in proximity to the right renal hilum, oriented parallel to the mesenteric arterial vasculature. Blunt dissection was employed to isolate the lymphatic vessel from surrounding adipose and connective tissues. A 25-gauge heparinized needle (pre-treated with 5% heparin sodium, 100 U/mL) was utilized to create an ostium in the lymphatic wall. Lymphatic effluents were collected via heparin-coated polyethylene tubing (PE-50, inner diameter 0.58 mm) connected to a calibrated micro collection system.

### 4.3. Hematoxylin and Eosin (H&E) Staining and Histopathological Score

Colonic specimens harvested from the distal region of rat intestines underwent fixation in 4% paraformaldehyde solution followed by paraffin embedding. Histological examination was performed on sectioned specimens using hematoxylin–eosin (H&E) dual staining protocol. Histomorphology assessment was conducted as previously described [33].

### 4.4. Magnetic Microbead-Based Cell Sorting

Following enzymatic dissociation under aseptic conditions, mononuclear cell suspensions were generated from mesenteric lymphatic tissues. Targeted lymphocytes were immunomagnetically labeled with anti-CD45R monoclonal antibodies (Miltenyi, Bergisch Gladbach, NRW, Germany). Subsequent magnetic-activated cell sorting (MACS) employing an OctoMACS separator coupled with a MACS Multi-Stand platform enabled the precise isolation of the CD45RA^+^ lymphocyte subpopulation. This standardized protocol achieved >95% purity, as verified by flow cytometric reanalysis. In the research, CD4^+^T cells were isolated from mesenteric lymph using CD4^+^ microbeads (Miltenyi, Bergisch Gladbach, NRW, Germany), with >95% purity confirmed by flow cytometry. Materials are shown in Table A1.

### 4.5. Mesenteric Lymphatic B Cell Adoptive Transfer and Intravital Imagination

Following labeling with DiR dye (Invitrogen, Carlsbad, CA, USA), 5 × 10^6^ mesenteric lymphatic B lymphocytes were intravenously administered through the caudal vein in a standardized adoptive transfer protocol. Images in vivo were acquired using an IVIS Spectrum (PerkinElmer, Waltham, MA, USA). Specifically, donor cells isolated from NC or TNBS rats were administered during the disease onset phase (days 6, 8, and 10 post-induction). Materials are shown in Table A1.

### 4.6. Bulk RNA Sequencing and Bioinformatic Analysis

MLB lymphocytes underwent RNA isolation, employing TRIzol Reagent (ThermoFisher, Waltham, MA, USA) with DNase I treatment. RNA sequence libraries were constructed and bioinformatic analysis was conducted as previously described [33]. Materials are shown in Table A1.

### 4.7. Real-Time Quantitative Polymerase Chain Reaction (RT-qPCR)

Quantitative real-time PCR was conducted according to a previously published protocol [33], and the rat primer sequences used in this study are listed as follows: *Gapdh*: forward primer: 5′-ACCCAGAAGACTGTGGATGG-3′, reverse primer: 5′-CACATTGGGGGTAGGAACAC-3′. *Ccl1*: forward primer: 5′-TTACAAAGAGATCGGCCCCAG-3′, reverse primer: 5′-TAAGGCGCAGCTTTCTCTACC-3′. *Cxcl11*: forward primer: 5′-TCCCCAAATAACACGAGGCA-3′, reverse primer: 5′-ATGTTTGCAGGGCACCTCAT-3′. *Cxcl12*: forward primer: 5′-ATGCCCCTGCCGATTCTTTG-3′, reverse primer: 5′-TGCACACTTGTCTGTTGTTGC-3′. *Mif*: forward primer: 5’-CGGACCGGGTCTACATCAAC-3′, reverse primer: 5′-ATAAACACAGAACGGGGCGT-3′. *Ccl17*: forward primer: 5′-GACCTTCGCCTGGACTTCTG-3′, reverse primer: 5′-ACTCTCGGCCTACATTGGTG-3′. *Ccl22*: forward primer: 5′-ACCACGTTTCGTGAAGGAGT-3′, reverse primer: 5′-CTGAGCCTTGTGGAGACACC-3′. *Cxcl13:* forward primer: 5′-TCCACCTCCAGGCAGAATGA-3′, reverse primer: 5′-TGGGTCTCCAGAATACCGTG-3′.

### 4.8. Immunocyte Isolation and Flow Cytometry

**Figure A1:**
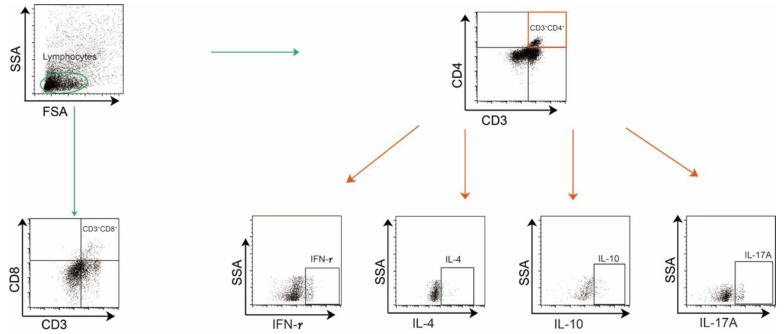
The gating strategy used to identify the CD3^+^CD4^+^ cells, CD3^+^CD8^+^ cells, IFN-γ^+^CD4^+^ T cells, IL-4^+^CD4^+^ T cells, IL-10^+^CD4^+^ T cells, and IL-17^+^CD4^+^ T cells in Figure 6 and Figure 7.

Colonic lamina propria mononuclear cells were enzymatically dissociated and mesenteric lymphocytes were isolated, as described previously [34]. Single-cell suspensions were stimulated with PMA/ionomycin and brefeldin A in RPMI-1640/10% FBS for 5 h at 37 °C/5% CO_2_ after Fcγ receptor blockade. Viability was assessed using Fixable Viability, followed by surface staining with anti-CD45 (Biolegend, San Diego, CA, USA), B220 (BD Biosciences, San Jose, CA, USA), CD3 (Biolegend, San Diego, CA, USA), CD4 (Biolegend, San Diego, CA, USA), and CD8 (eBioscience, San Diego, CA, USA) (in PBS/0.5% BSA at 4 °C. Intracellular cytokines IL-10 (BD Biosciences, San Jose, CA, USA), IFN-γ (Biolegend, San Diego, CA, USA), IL-4 (Invitrogen, Carlsbad, CA, USA), IL-17 (Invitrogen, Carlsbad, CA, USA), were detected via clone-specific antibodies. Fluorescence minus one (FMO) control, unstained, and single-color control samples were used to determine cytokine cutoffs for negative and positive assays and calculate compensation. The gating strategies are shown in Figure A1.

### 4.9. Knockdown of Ccl1 Expression in Colonic CD4^+^ T Cells

CD4^+^ T lymphocytes isolated from colonic tissues of normal control and TNBS-induced colitis rats via CD4^+^ microbead-based magnetic sorting were cultured in RPMI-1640 medium supplemented with 10% FBS, dual antibiotics (100 U/mL penicillin–streptomycin), 3 μg/mL soluble CD28 antibody (eBioscience, San Diego, CA, USA), and pre-coated CD3 mAb (5 μg/mL) under 37 °C/5% CO_2_ conditions. Three *Ccl1*-targeting siRNA duplexes (si-*Ccl1*-1: 5′-GCUUGAACACCUUGGAGAA-3′/5′-UUCUCCAAGGUGUUCAAGC-3′; si-*Ccl1*-2: 5′-GUUUAUCAAAUGUUACAAA-3′/5′-UUUGUAACAUUUGAUAAAC-3′; si-*Ccl1*-3: 5′-GUUCAAGAUUACCUGAAGA-3′/5′-UCUUCAGGUAAUCUUGAAC-3′) were complexed with cationic lipids (1:2 *w*/*w* in 5% glucose) and transfected (50 nM) into 60–70%-confluent cells using antibiotic-free medium for 24 h. Quantitative PCR validation demonstrated >80% transcript suppression, with si-*Ccl1*-3 showing superior efficacy (60–70% reduction vs. scrambled controls, *p* < 0.01), qualifying it for functional analyses. Materials are shown in Table A1.

### 4.10. Co-Culture of Colonic CD4^+^ T Cells and MLB Cells

For migration analysis, isolated colonic CD4^+^ T cells were seeded in 24-well glass-bottom chambers at 5 × 10⁴ cells/well and transfected with 50 nM si-*Ccl1*-3 for 24 h in RPMI 1640/10% FBS. A Transwell co-culture system was established by placing sorted MLB cells (1 × 10⁵ cells/insert) from NC or TNBS rats into the upper chamber. The experimental groups included the following: TNBS MLB+NC T, TNBS MLB+TNBS T, TNBS MLB+NC T-*Ccl1* KD, and TNBS MLB+TNBS T-*Ccl1* KD, with 3~6 technical replicates. Live-cell imaging was performed using a Leica THUNDER Imager (Leica Microsystems, Wetzlar, Germany) with an incubation chamber (37 °C, 5% CO_2_) from 12 to 24 h post-co-culture, capturing time-lapse videos (1 frame/10 min) using LAS X software (v3.7.4). MLB cells exhibited characteristic lymphocyte morphology (diameter of 6–8 μm, phase-bright) distinct from epithelial IEC-6 cells (diameter of 15–20 μm). Migration quantification involved the random selection of four 200× fields per well, with CD19^+^ B cells in lower chambers counted using ImageJ (v1.53t) particle analysis after background subtraction (rolling ball radius of 50 pixels). Data were normalized to control group migration indices.

For differentiation analysis, immunoassay plates were coated with anti-CD3 monoclonal antibody (5 μg/mL) in PBS at 4 °C for 16 h, washed thrice with DPBS, and then loaded with 5 × 10^6^ cells (MLB cells: colonic CD4^+^ T cells = 1:2) in RPMI 1640 medium supplemented with 10% FBS, 100 U/mL penicillin–streptomycin, anti-CD28 (3 μg/mL), and anti-CD40 (10 μg/mL), followed by 24 h incubation under standard conditions (37 °C, 5% CO_2_, 95% humidity). Materials are shown in Table A1.

### 4.11. Statistical Analysis

Quantitative data are expressed as means ± standard error of measurement (SEM). Parametric comparisons were conducted using a two-tailed unpaired Student’s *t*-test (for two groups) or one-way analysis of variance (ANOVA) with Tukey’s post hoc analysis (≥3 groups). Nonparametric datasets underwent a Mann–Whitney U test (two groups) or a Kruskal–Wallis test with Dunn’s correction (multiple groups). Statistical thresholds were set at *p* < 0.05 (*), *p* < 0.01 (**), *p* < 0.001 (***), and *p* < 0.0001 (****). All analyses were executed in GraphPad Prism v9.5.0 (GraphPad Software, La Jolla, CA, USA).

## 5. Conclusions

The present study revealed that both the number and function of MLB cells were altered in TNBS-induced colitis rats. MLB cells exhibited a tendency to migrate to the intestine and exacerbate colitis through the Ccr8-Ccl1 axis, potentially by contributing to intestinal T cell-mediated inflammation.

## Data Availability

The original contributions presented in the study are included in the article. Further inquiries can be directed to the corresponding authors.

## Abbreviations

The following abbreviations are used in this manuscript:

B220	Protein Tyrosine Phosphatase Receptor Type C
CD	Crohn’s Disease
CD3	Cluster of Differentiation 3
CD4	Cluster of Differentiation 4
CD8	Cluster of Differentiation 8
CD14	Cluster of Differentiation 14
CD28	Cluster of Differentiation 28
CD32	Cluster of Differentiation 32
CD40L	Cluster of Differentiation 40 Ligand
CD45	Cluster of Differentiation 45
CD45RA	Cluster of Differentiation 45RA
CD86	Cluster of Differentiation 86
Cd19	Cluster of Differentiation 19
Cd27	Cluster of Differentiation 27
Cd40	Cluster of Differentiation 40
Cd79b	Cluster of Differentiation 79b
Ccl1	C-C Motif Chemokine Ligand 1
Ccl17	C-C Motif Chemokine Ligand 17
Ccl22	C-C Motif Chemokine Ligand 22
Ccr4	C-C Motif Chemokine Receptor 4
Ccr8	C-C Motif Chemokine Receptor 8
Cxcr4	C-X-C Motif Chemokine Receptor 4
Cxcr5	C-X-C Motif Chemokine Receptor 5
Cxcl11	C-X-C Motif Chemokine Ligand 11
Cxcl12	C-X-C Motif Chemokine Ligand 12
Cxcl13	C-X-C Motif Chemokine Ligand 13
DSS	Dextran Sodium Sulfate
DiR	1,1-Dioctadecyl-3,3,3,3-tetramethylindotricarbocyaineiodide
DCs	Dendritic Cells
FMO	Fluorescence Minus One
GSEA	Gene Set Enrichment Analysis
GALT	Gut-Associated Lymphoid Tissue
IBD	Inflammatory Bowel Disease
IL-4	Interleukin 4
IL-10	Interleukin 10
IL-17	Interleukin 17
IFN-γ	Interferon-gamma
Mif	Macrophage Migration Inhibitory Factor
ML	Mesenteric Lymphatics
MLB	Mesenteric Lymphatic B
MLNs	Mesenteric Lymph Nodes
NC	Normal Control
SD	Sprague Dawley
TLOs	Tertiary Lymphoid Organs
TNBS	2,4,6-Trinitrobenzenesulfonic Acid
TNF-α	Tumor Necrosis Factor-alpha
UC	Ulcerative Colitis
VEGF	Vascular Endothelial Growth Factors
VEGFR-3	Vascular Endothelial Growth Factor Receptor 3
VEGF-C	Vascular Endothelial Growth Factor C

## Appendix A

**Table A1:** List of materials and equipment.

Materials and Equipment	Manufacturer/Country	Catalog Number
CD45R MicroBeads	Miltenyi Bergisch Gladbach, NRW, Germany	130-090-494
CD4 MicroBeads	Miltenyi Bergisch Gladbach, NRW, Germany	130-090-319
DiR	Invitrogen, Carlsbad, CA, USA	D12731
CD45	Biolegend, San Diego, CA, USA	202214
B220	BD Biosciences, San Jose, CA, USA	743590
CD3	Biolegend, San Diego, CA, USA	201403
CD4	Biolegend, San Diego, CA, USA	201520
CD8	eBioscience, San Diego, CA, USA	56-0084-82
IL-10	BD Bioscience, San Jose, CA, USA	555088
IFN-γ	Biolegend, San Diego, CA, USA	507806
IL-4	Invitrogen, Carlsbad, CA, USA	50-7045-82
CD32	BD Bioscience, San Jose, CA, USA	550271
TRIzol	ThermoFisher, Waltham, MA, USA	15596018
IL-17	Invitrogen, Carlsbad, CA, USA	48-7177-82
Anti-CD3 antibody	eBioscience, San Diego, CA, USA	16-0030-85
Anti-CD28	eBioscience, San Diego, CA, USA	16-0280-85
Anti-CD40	Invitrogen, Carlsbad, CA, USA	16-0402-81
TNBS	Sigma, St. Louis, MO, USA	P2297
si*Ccl1* 1~3	Tsingke Biotech, Beijing, China	/
IVIS Spectrum	PerkinElmer, Waltham, MA, USA	/
NanoPhotometer spectrophotometer	IMPLEN GmbH, Munich, Germany	/
Bioanalyzer 2100 system	Agilent Technologies, Santa Clara, CA, USA	/
GraphPad Prism v9.5.0	GraphPad Software, La Jolla, CA, USA	/
